# LncRNA MIR17HG Suppresses Breast Cancer Proliferation and Migration as ceRNA to Target FAM135A by Sponging miR-454-3p

**DOI:** 10.1007/s12033-023-00706-1

**Published:** 2023-03-21

**Authors:** Jingjing Xu, Meishun Hu, Yang Gao, Yishu Wang, Xiaoning Yuan, Yan Yang, Wenjing Song, Weinan Yin, Pengju Gong, Lei Wei, Jingwei Zhang

**Affiliations:** 1 Department of Breast and Thyroid Surgery, Zhongnan Hospital, Hubei Key Laboratory of Tumor Biological Behaviors, Hubei Cancer Clinical Study Center, Wuhan University, Wuhan, 430071 Hubei China; 2 Department of Pathology and Pathophysiology, Hubei Provincial Key Laboratory of Developmentally Originated Disease, School of Basic Medical Sciences, Wuhan University, Wuhan, 430071 Hubei China; 3 Department of Gastrointestinal Surgery & Department of Gastric and Colorectal Surgical Oncology, Zhongnan Hospital of Wuhan University, No. 169 Donghu Road, Wuchang District, Wuhan, 430071 China; 4 Hubei Key Laboratory of Tumor Biological Behaviors, No. 169 Donghu Road, Wuchang District, Wuhan, 430071 China; 5 Hubei Cancer Clinical Study Center, No. 169 Donghu Road, Wuchang District, Wuhan, 430071 China; 6 Department of Legal English and TOEIC, The University of Adelaide, North Terrace, 5005 Australia

**Keywords:** Breast cancer, lncRNA, ceRNA, MIR17HG, miR-454-3p, FAM135A

## Abstract

Breast cancer is one of the most common malignant tumors in women, and causes a large number of cancer-related deaths. The main cause of death of breast cancer patients is tumor recurrence and metastasis. Recent studies show that lncRNA (Long non-coding RNA) plays an important role in breast cancer. However, the overall biological activity and clinical consequences of the lncRNA MIR17HG in breast cancer remain unclear. Thus, we investigate how the MIR17HG/miR-454-3p network impacts breast cancer cell proliferation and migration. Given the TCGA and Oncomine databases, the researchers evaluated variations in MIR17HG expression for the survival rates of breast cancer patients. The influence of MIR17HG on cell proliferation, migration, cell cycle, and the mRNA expression level of miR-454-3p and FAM135A (family with sequence similarity 135 member A) is identified. Luciferase assay was used to detect the regulatory effect of miR-454-3p on the 3′UTR region of FAM135A, and rescue experiments demonstrated that MIR17HG can up-regulate FAM135A expression by competitively binding miR-454-3p. The effect of FAM135A on the cloning and invasion of MCF-7 cells was detected. MIR17HG expression is reduced in breast cancer tissues, and patients with greater levels of MIR17HG expression have a better prognosis. MIR17HG overexpression caused G2/M arrest in breast cancer cells according to a flow cytometry assay. FAM135A knockdown enhances breast cancer cell proliferation and clone creation, as well as two-dimensional and three-dimensional migratory capacities. Patients with high FAM135A expression in their breast cancer had a better prognosis. These novel findings indicate that MIR17HG may be a potential target for breast cancer. Our findings demonstrated that MIR17HG might suppress breast cancer cell proliferation and migration by sponge miR-454-3p through ceRNA(competing endogenous RNAs) mechanism, indicating that targeting MIR17HG may be a feasible therapeutic candidate for breast cancer.

**Graphical Abstract:**

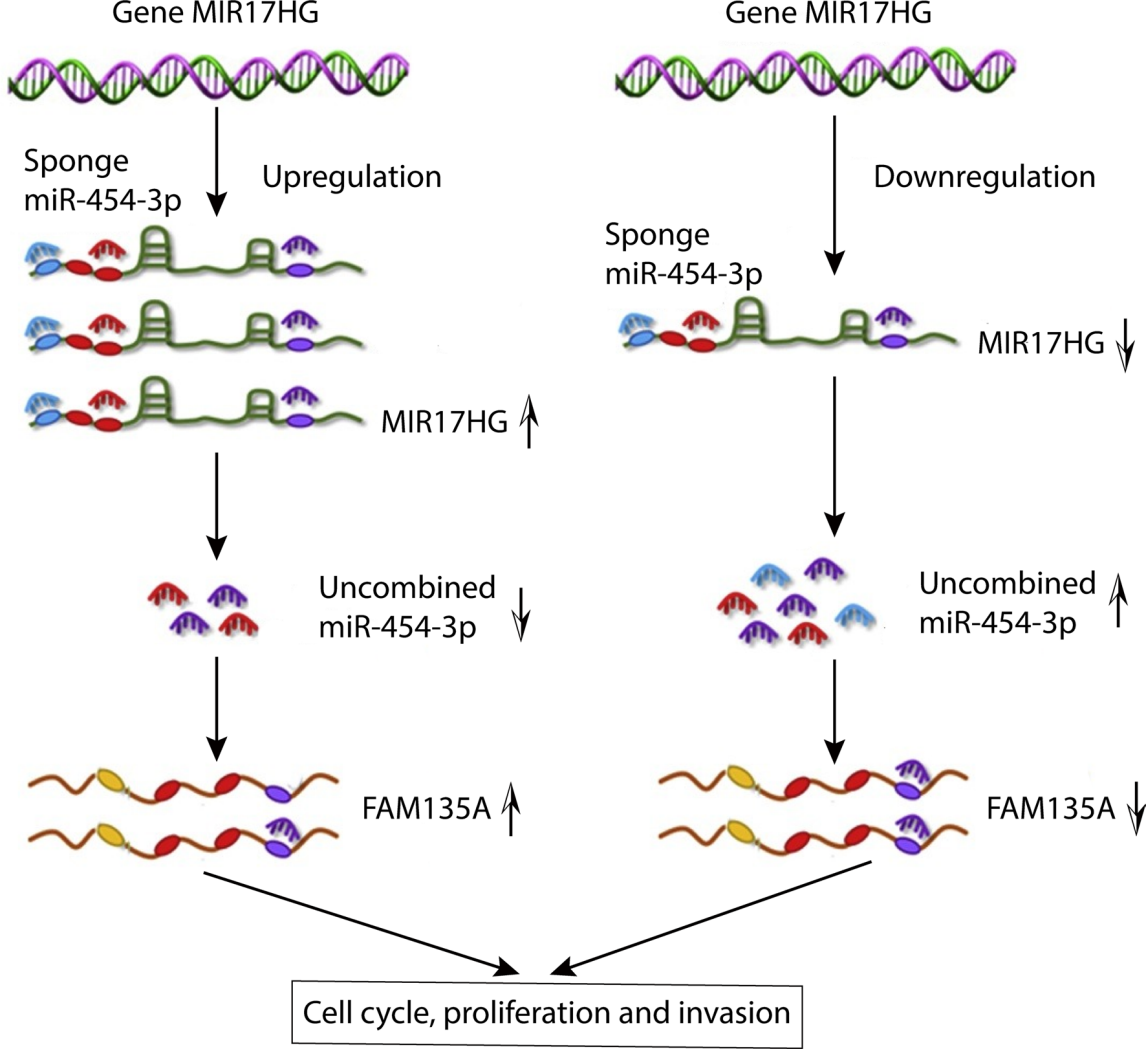

## Introduction

Breast cancer is one of the most frequent cancers in women [1], accounting for a large number of cancer-related deaths each year [2]. The main cause of death of breast cancer patients is tumor recurrence and metastasis. Breast cancer is a complicated illness that develops as a result of a multifactorial process that can be triggered by changes in gene expression or environmental variables. Therefore, it is important to study the underlying mechanism of breast metastasis and explore new targets for breast cancer therapy.

LncRNA is a novel form of RNA transcript that is longer than 200 nucleotides but does not have the potential to code for proteins [3, 4]. Despite being classified as transcription “noise” [5], research shows that lncRNAs is of great importance in a variety of tissues and cell types [6, 7]. Current study indicates lncRNA plays a crucial role in the development of breast cancer. The role of the majority of lncRNAs in breast cancer, on the other hand, is mostly unknown [8, 9]. Previous studies have found that lncRNAs function as scaffolds of RNP complexes, decoys of transcription factors or miRNAs, RNA interference, transcription factors, or chromatin‐modifying proteins to target specific genomic sites, and cis‐ or trans‐transcriptional regulation, thus playing a role in tumor development [10].There are studies that have shown that lncRNA can directly regulate the expression of neighboring genes through the cis‐acting mechanism, which constitute a substantial fraction of lncRNAs with an attributed function, regulate gene expression in a manner dependent on the location of their own sites of transcription, at varying distances from their targets in the linear genome [4]. However, the regulation between lncRNA and target genes in ceRNA regulation mode is indirect and mutual. CeRNAs are transcripts that can regulate each other at post-transcription level by competing for shared miRNAs. CeRNA networks link the function of protein-coding mRNAs with that of non-coding RNAs such as microRNA, long non-coding RNA, pseudogenic RNA and circular RNA [10]. According to previous research, lncRNA can compete with endogenous RNA (ceRNA) in a way that permits microRNA (miR) to govern cancer cell proliferation, migration, invasion, and death [11]. MicroRNA-RISC complexes, which contain the protein Argonaute 2 (AGO2), can target certain mRNAs via complementarity with the mRNA’s 3′-untranslated region (UTR). This interaction, in turn, impairs the stability and/or translation of the target mRNA. As a result, it is crucial to identify key oncogenic lncRNAs for tumor diagnosis and therapy [8].

In previous study, our group found that the lncRNA-miRNA-mRNA network could play a critical role in mediating breast cancer [12], and we analyzed that MIR17HG has low-expression and improve the prognosis in The Cancer Genome Atlas breast cancer tissue high‐throughput sequencing data. The MIR17HG gene is derived from the mir-17-92a-1 cluster host gene on chromosome 13q31 (also known as c130rf25). The c13orf25 transcript produces six functional miRNAs in addition to the lncRNA MIR17HG: miR-17, miR-18a, miR-19a, miR-20a, miR-19b-1, and miR-92a-1 [13]. This region is thought to be crucial in cell survival, proliferation, differentiation, and angiogenesis, and it has been linked to development of many types of human cancer [14]. MIR17HG family members are found in high concentrations in aggressive malignancies such as metastatic colorectal cancer, retinoblastoma, and pancreatic cancer [15]. MIR17HG is an oncogene that is transcribed as LncRNA, but it does not encode a protein. The rs4824505 allele and AC haplotype of the MIR17HG gene’s rs4824505/rs7336610 subtype were shown to be related with a higher risk of developing breast cancer [16]. However, little is known about the expression of the lncRNA MIR17HG and its potential role in breast cancer.

In previous study, miRNAs play strong regulatory roles in various tumor processes. miRNAs are small (19–24nt) non-coding regulatory RNAs. They act as post-transcriptional regulators of gene, binding complementarily to the target mRNA and resulting in the arrest of translation or mRNA degradation [17]. miRNAs have been deemed as promising invasive cancer biomarkers for advanced diagnosis and prognosis [18].

According to StarBase (http://starbase.sysu.edu.cn/), MIR17HG has a highly linked potential binding to miR-454-3p, and FAM135A has a putative binding site for miR-454-3p, indicating that MIR17HG may influence FAM135A expression in breast cancer cells. Therefore, we hypothesized that MIR17HG also regulates the development of breast cancer cells and thus affects the prognosis of breast cancer patients through the ceRNA mechanism. Then we found that LncRNA MIR17HG competes with FAM135A for miR-454-3p binding, therefore abolishing the suppressive effect of miR-454-3p on FAM135A. Consistent with the previous assumption, our data demonstrated MIR17HG/miR-454-3p/FAM135A axis functions aslncRNA-miRNA-mRNA network through ceRNA mechanism in breast cancer.

FAM135A, also known as KIAA1411, is a gene in the RP25 locus that is situated between D6S1557 and D6S421. FAM135A has a 6280 bp transcript and a 1541 amino acid long translated protein. FAM135A is expressed in the retina, brain, skeletal muscle, pancreas, and lung, according to gene expression studies. The homo gene shares around 84 percent of its nucleic acid sequence with rat and mouse homologue genes, indicating that the protein has conserved activities in both mammals and that the gene may represent an extremely rare single nucleotide polymorphism [19].

The goal of this research is to look at the interaction between MIR17HG and FAM135A expression in breast cancer cells, as well as the link between MIR17HG expression and breast cancer cell proliferation and migration, as well as possible mechanism. We propose that the LncRNA MIR17HG and FAM135A compete for the miRNA miR-454-3p, and decreasing MIR17HG increases the binding of miR-454-3p and FAM135A, leading to the inhibition of FAM135A mRNA binding for its expression or direct degradation. FAM135A is linked to several tumor-related pathways and is strongly correlated with breast cancer cell invasion and migration, according to extensive bioinformatics and experimental studies. This study will help clarify the mechanism of LncRNA MIR17HG in the incidence and development of breast cancer, as well as future research on the molecular mechanism of FAM135A in breast cancer invasion and metastasis. This study will also serve as a foundation for future research into the molecular theoretical processes of breast cancer development and metastasis.

In this study, we discovered that MIR17HG/miR-454-3p/FAM135A axis functions in preventing development and metastasis in breast cancer. These novel findings indicate that MIR17HG/miR-454-3p/FAM135A axis may be a potential target for breast cancer therapy to improve the survival time and quality of life of patients.

## Materials and Methods

**Table:** 

Materials and Chemicals	Manufacturers
HG-DMEM	Gibco, Italy
FBS	Gibco, Italy
Penicillin–Streptomycin Solution	Hyclone, USA
Lipofectamine 2000	Zoman Biotechnology Co., Ltd., China
T4 DNA Ligase	Sevier Biotechnology Co., Ltd., China
Restriction Enzyme AgeI SpeI	Thermo Fisher Scientific, USA
Agarose	Bowest, Spain
DNA Marker	Thermo Fisher Scientific, USA
Agarose Gel DNA Extraction Kit	Tsingke Biotechnology Co., Ltd., China
Cell Freezing Medium	New Cell & Molecular Biotech Co., Ltd
Ampicillin	Oxiod, the UK
Tryptone	Oxiod, the UK
Yeast Extract	Oxiod, the UK
Plasmid Extraction Kit	Tsingke Biotechnology Co., Ltd., China
Dual Luciferase Reporter Gene Assay Kit	Yeasen Biotechnology Co., Ltd., China
SYBR Green qPCR mix	Sevier Biotechnology Co., Ltd., China
First Strand cDNA Synthesis Kit	Sevier Biotechnology Co., Ltd., China
Transwell Chamber	Corning, USA

All other reagents were analyzed and purified by Sinopharm Group Chemical Reagent Co., LTD.

### Cell Culture

The MCF-7 cells and HEK293T cells were purchased from the China Center for Type Culture Collection (CCTCC, Chinese Academy of Sciences, Shanghai, China), were cultured in medium (DMEM, HyClone, USA, SH30022.01B) supplemented with 10% fetal bovine serum (FBS) (Gibco, Milano, Italy, 10099-141), culture conditions: temperature: 37 °C; 5% CO_2_.

### Plasmids and Cell Transfection

To meet the experimental requirements, the FAM135A 3′UTR sequence with the full length of the FAM135A 3′UTR region and the miR-454-3p binding site mutation was constructed into pMIR-report-luci vector and the restriction sites were SpeI and SacI. pMIR-report-FAM135A-3′UTR WT(forward: 5′-CTAGTACTTGTGAAAATAAAAATGCACTATACTTGTGAAAATAAAAATGCACTATACTTGTGAAAATAAAAATGCACTAAGCT-3′; reverse: 5′-TAGTGCATTTTTATTTTCACAAGTATAGTGCATTTTTATTTTCACAAGTATAGTGCATTTTTATTTTCACAAGTA-3′), pMIR-report-FAM135A-3′UTR mut(forward: 5′-CTAGTAGTAGAGATTATTTATAACGTGAATAGTAGAGATTATTTATAACGTGAATAGTAGAGATTATTTATAACGTGAAAGCT-3′, reverse: 5′-TTCACGTTATAAATAATCTCTACTATTCACGTTATAAATAATCTCTACTATTCACGTTATAAATAATCTCTACTA-3′). The shRNAs (short hairpin RNAs) of MIR17HG:sh1-Forward: 5′-CCGGGGTGGCCTGCTATTTCCTTCACTCGAGTGAAGGAAATAGCAGGCCACCTTTTTG-3′, sh1-Reverse: 5′-AATTCAAAAAGGTGGCCTGCTATTTCCTTCACTCGAGTGAAGGAAATAGCAGGCCACC-3′, sh2-Forward: 5′-CCGGGTCTAACTACAAGCCAGACTTCTCGAGAAGTCTGGCTTGTAGTTAGACTTTTTG-3′, sh2-Reverse: 5′-AATTCAAAAAGTCTAACTACAAGCCAGACTTCTCGAGAAGTCTGGCTTGTAGTTAGAC-3′,

The shRNAs (short hairpin RNAs) of FAM135A:sh1-Forward: 5′-CCGGGGGCGAGAGGGTTGAAGATCACTCGAGTGATCTTCAACCCTCTCGCCCTTTTTG-3′,sh1-Reverse: 5′-AATTCAAAAAGGGCGAGAGGGTTGAAGATCACTCGAGTGATCTTCAACCCTCTCGCCC-3′,sh2-Forward: 5′-CCGGGCGAGAGGGTTGAAGATCAAC CTCGAGGTTGATCTTCAACCCTCTCGCTTTTTG-3′,sh2-Reverse: 5′-AATTCAAAAAGCGAGAGGGTTGAAGATCAAC CTCGAGGTTGATCTTCAACCCTCTCGC-3′,sh3-Forward: 5′-CCGGGTTTGCGGTTGCTGTGATGGCCTCGAGGCCATCACAGCAACCGCAAACTTTTTG-3′,sh3-Reverse: 5′-AATTCAAAAAGTTTGCGGTTGCTGTGATGGC CTCGAGGCCATCACAGCAACCGCAAAC-3′. These plasmids were all sequenced and verified.

miR-454-3p mimics, and miR-454-3p inhibitors were obtained from GenePharma in Shanghai for MIR17HG or FAM135A negative controls (B02001, B03001). Inoculate HEK293T cells at a density of 1 × 10^5^ in a 24-well plate and harvest 60–80% density the next day.Then, using Lipofectamine 2000 reagent, shRNAs and plasmids were transfected (Invitrogen Co, Ltd. 11668-027). The technique for transfection is described in the instruction manual. Cells were collected 48 h after transfection to determine the biological impact or degree of target gene expression.

### RNA Isolation and Real-Time Quantitative PCR

The Trizol reagent was used to extract total RNA from cells (Invitrogen, Carlsbad, CA, 15596-026). cDNA was created using the GeneAmp™ RNA PCR Core Kit’s first-strand cDNA synthesis (Thermo Scientific, USA, K1622). qPCR was used to detect and evaluate the amount of mRNA expression (Applied Biosystem Inc.). For relative expression analysis, the 2^−ΔΔCt^ formula algorithm is employed. The sequences of the primers are as follows:

U6-F: 5′-GCTTCGGCAGCACATATACTAA-3′, U6-R: 5′-CGAATTTGCGTGTCATCCTT-3′miR-454-3p-RT Primer: 5′-GTCGTATCCAGTGCAGGGTCCGAGGTATTCGCACTGGATACGACACCCTA-3′miR-454-3p-Fp: 5′-AACACGCTAGTGCAATATTGCT-3′miR-454-3p-Rp: 5′-GTCGTATCCAGTGCAGGGT-3′, GAPDH-F: 5′-GCACCACCAACTGCTTA-3′, GAPDH-R: 5′-AGTAGAGGCAGGGATGAT-3′, (q)MIR17HG-F: 5′-AGCAGTAAAGGTAAGGAGAGC-3′, (q)MIR17HG-R: 5′-CTGAAGTCTCAAGTGGGCAT-3′, (q)FAM135A-F: 5′-GCACTACACCAGCCACTAATA-3′, (q)FAM135A-R: 5′-CAAGAGTAGGAATCACGGAATCT-3.

When the reaction system is well prepared, put it on the Fluorescence quantitative PCR (BIO-RAD, CFX96, 15000193, Singapore) and run it.

### Luciferase Assay

pMIR-report-FAM135A WT and mut were co-transfected into HEK293T cells with Lipofectamine 2000 and miR-454-3p NC inhibitor or miR-454-3p inhibitor, with PLR-TK (Promega, USA) as a control. Transfection occurred at a rate of 10:30:1. The relative luciferase activities were measured 48 h after transfection using a Dual-Luciferase reporter Assay Kit (Promega, Madison, WI, USA, E1910) and a GloMax 20/20 Luminometer (Promega, E5311) according to the manufacturer’s instructions.

### Flow Cytometry to Detect Cell Cycle

The cell cycle was measured using flow cytometry. After being transfected for 48 h, the cells were starved for 12 h. The medium was changed to a serum-containing medium for 8 h. The serum was halted for digestion after 45 s of trypsin digestion and fixed with pre-cooled anhydrous ethanol. After the ethanol was removed, each tube was filled with PBS and centrifuged at 4 °C. The supernatant was removed and the cells was incubated with the staining solution (the PI staining solution should be incubated for 30 min at 37 °C in the dark). After incubation, each tube was centrifuge at 4 °C, the supernatant was removed, and each tube was resuspended in PBS. The fluorescence intensity was assessed using flow cytometry (BD Biosciences, USA), and the cytometer model must be selected as a flow cytometer software (BD FACSDiva 7.0).

### Cell Proliferation Assay

The Cell Counting Kit 8 was used to measure cell proliferation (CCK-8; Beyotime, Jiangsu Province). For the CCK-8 experiment, cells (1 × 10^3^cells/well) were seeded on 96-well plates (Cell Counting Kit-8, Zomanbio, Beijing, ZP328-2). After 0, 12, 24, and 48 h of growth, the cells were administered the CCK-8 solution. The OD450 value was determined using an automated microplate reader after 2 h of incubation (Bio-Tek, USA, FLx800). On 6-well plates, 300 cells were planted per well for the colony formation experiment. The cells were fixed for 30 min with 4% paraformaldehyde and stained for 30 min with 0.1% crystal violet before being counted using Image J around 2 weeks later.

### Cell Migration Ability Determination

To begin, we used a ruler and a marker to draw three equal lines on the back of the six-well plate. After scratching the cells, rinsing them in PBS, and adding 5% serum media, the cells were imaged under a light microscope (100 ×) using ScopeTek software after 0, 24, and 48 h of incubation. Image J was used to determine the distance traveled by cells into the scratched region.

### Transwell Detection of Cell Migration and Invasion

A 24-well Transwell chamber polyvinyl-pyrrolidone-free polycarbonate filter (8 m pore size) was used in the Transwell assay to assess migration. The lower chamber was filled with 500 μl DMEM containing 10% FBS, and the top chamber with serum-free DMEM containing 2 × 10^4^cells. After 24 h, cells were fixed and stained with 4% paraformaldehyde and 5% crystal violet, photographed with a ScopeTek under a light microscope (100 ×), and the cell number was counted with Image J.

### Western Blotting

The protein was extracted with RIPA lysis buffer, and quantified with BCA Protein Assay Kit. Preparing polyacrylamide gel with 8% separation gel and adding the protein sample to the sample hole with 20 μg. The protein was bound to the PVDF membrane by electrophoretic and wet transmembrane with running buffer and transfer buffer. After blocking the nonspecific sites on the membrane with 5% skim milk powder at room temperature about 2 h. And then incubate the PVDF membrane in a primary antibody of GAPDH and FAM135A for 8 h at 4 °C. The membrane was incubated with the corresponding secondary antibody for 1 and a half h at room temperature, and finally detected by ECL reagents and digital imager. The optical density of bands was measured by a computer-assisted imaging analysis system.

### Statistical Analysis

For statistical analysis, the GraphPad Prism 7 program was utilized. Each experiment was repeated 3 times. The data were given as the mean ± standard deviation (SD). The *t* test was performed to examine the difference between two groups. *p* < 0.05 was considered statistically significant.

## Results

### MIR17HG is Down-Regulated in Breast Cancer Tissue

**Fig. 1:**
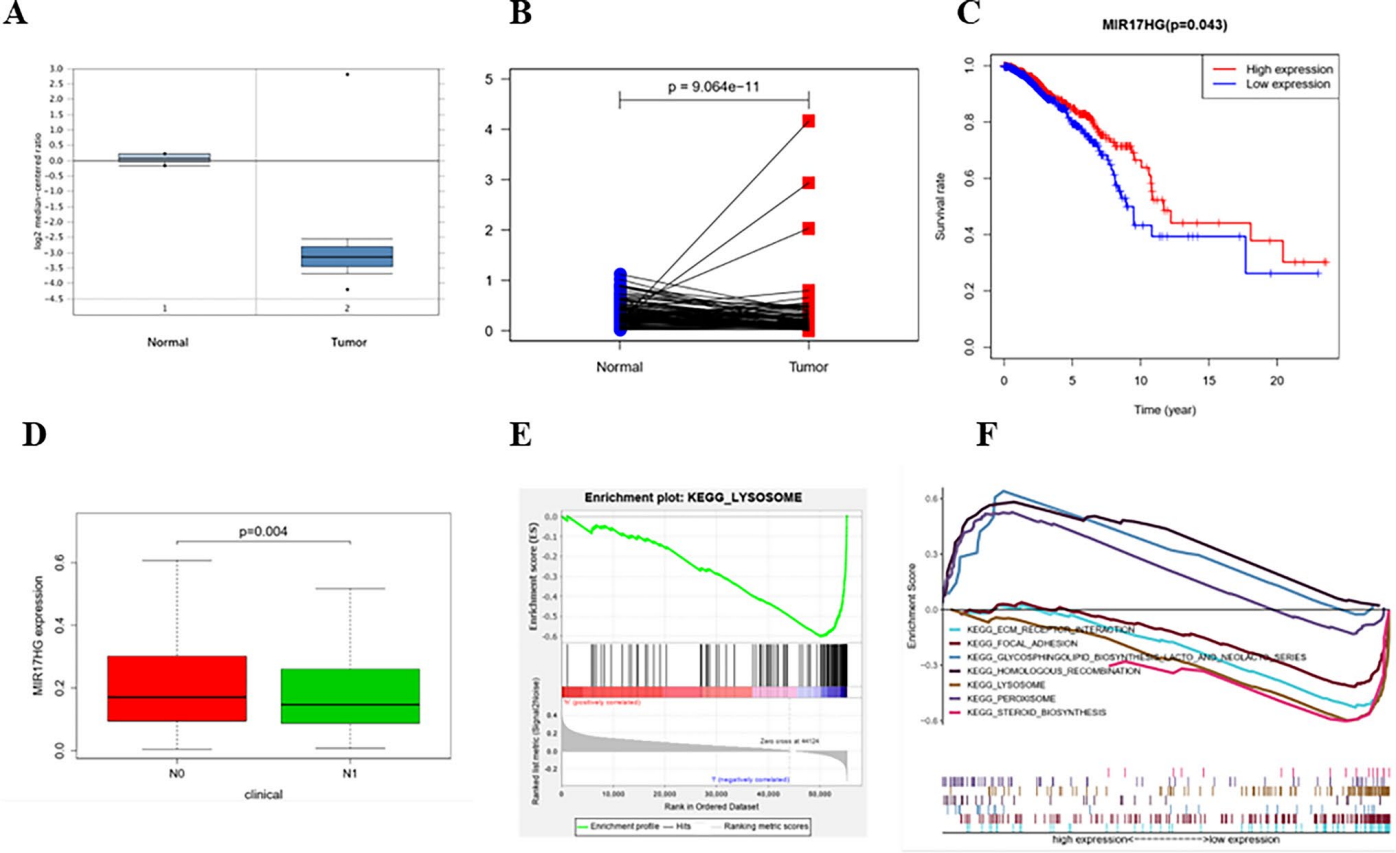
MIR17HG is down-regulated in breast cancer tissue. The differential expression of MIR17HG in breast cancer samples and adjacent normal breast tissues is shown, according to Oncomine (**A**) and TCGA (**B**) database. (**C**) The relationship of MIR17HG with survival rate of breast cancer patients is analyzed in TCGA database by R language. (**D**) The differential expression of MIR17HG in N0 breast cancer samples and N1 breast cancer samples in TCGA database. (**E**) Pathways in lysosome. (**F**) Pathways in ECM receptor interaction, focal adhesion, glycosphingolipid biosynthesis lacto and neolacto series, homologous recombination, lysosome, peroxisome, steroid biosynthesis

The expression of MIR17HG in breast cancer tissue was investigated to see if it plays a role in the disease. MIR17HG expression was shown to be lower in human breast cancer tissues as compared to the control group (Fig. 1A). MIR17HG levels in 593 pairs of breast cancer and paracancerous tissues were assessed in the Oncomine database. When compared to the normal control group, the expression of MIR17HG in most breast cancer tissues was much lower (Fig. 1B). In order to investigate the association between the expression level of MIR17HG and the clinicopathological characteristics of breast cancer patients, the TCGA database was utilized to evaluate the link between the expression level of MIR17HG and the survival rate of breast cancer patients. The data demonstrate that patients with breast cancer who have high MIR17HG expression have a higher survival rate, whereas patients with low MIR17HG expression have a lower survival rate, and the difference is statistically significant (*p* < 0.05) (Fig. 1C). The expression level of MIR17HG in breast cancer patients in N0 and N1 were compared based on the involvement of regional lymph nodes. The level of MIR17HG expression in breast cancer patients is linked to lymph node metastasis (Fig. 1D). According to the findings of the GSEA(Gene Set Enrichment Analysis), the low expression of MIR17HG has a relatively strong link to lysosome-related pathways (Fig. 1E). Low MIR17HG expression correlates strongly with ECM receptor interaction, focal adhesion, steroid biosynthesis, and lysosome pathways, whereas high MIR17HG expression correlates strongly with glycosphingolipid biosynthesis lacto and neolacto series, peroxisome, and homologous recombination (Fig. 1F).

### Knockdown of MIR17HG can Promote Proliferation and Metastasis in Breast Cancer Cells

**Fig. 2:**
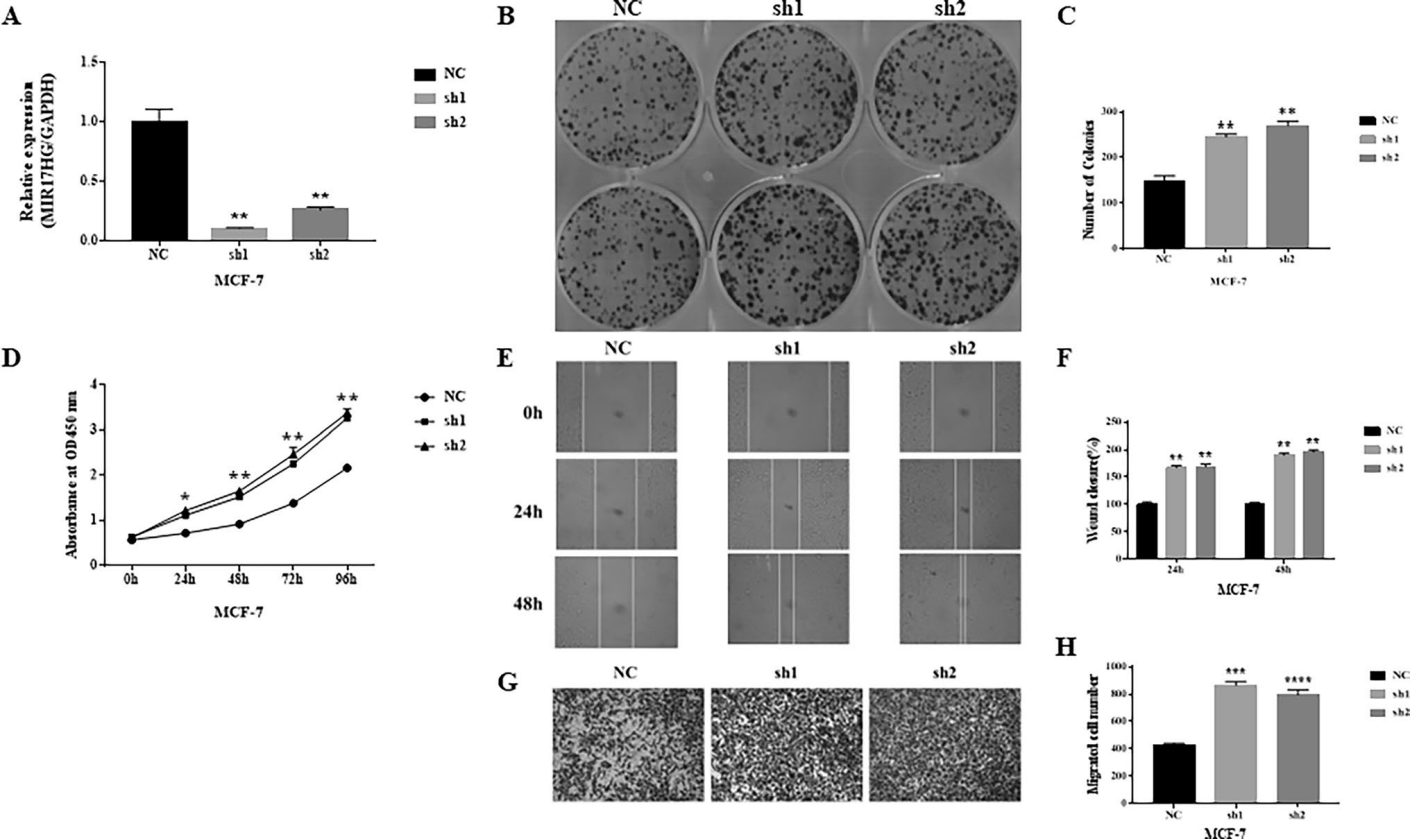
Knockdown of MIR17HG can promote proliferation and metastasis in breast cancer cells. (**A**) The expression of MIR17HG is significantly decreased by transfection of MIR17HG shRNA in MCF-7 cells, which is detected by qRT-PCR. (**B**, **C**) Colony formation assays show that knockdown of MIR17HG inhibits breast cancer cell proliferation. (**D**) Growth curves of MCF-7 cells after transfection with sh-MIR17HG or sh-NC is determined via CCK-8 assays. (**E**, **F**) shMIR17HG results in a quicker closing of scratch wound in MCF-7 cells by wound healing assay. (**G**, **H**) Transwell migration assay is measured and the results are expressed as the number of invaded cells per field

The knockdown effectiveness of two shRNAs designed to knock down MIR17HG in breast cancer cells is evaluated using qPCR, which reveals that both MIR17HG shRNAs are successful (Fig. 2A). CCK8 assays are performed when MIR17HG is knocked down in MCF-7 cells. We find MIR17HG knockdown considerably improves MCF-7 cell growth (Fig. 2D). Colony formation studies illustrate that knocking down MIR17HG substantially increases the number of colonies, which is consistent with CCK8 assay results (Fig. 2B, C). Wound healing assays are subsequently performed and showed that wound closure occurred in MCF-7 transfected with shMIR17HG as well as in the control group compared to the NC group, but wound closure is more pronounced in the former group (Fig. 2E, F). Using Transwell assays, we then look at how MIR17HG affects breast cancer cell migration. According to the data, MIR17HG downregulation significantly aided MCF-7 migration (Fig. 2G, H).

### Overexpression of MIR17HG Induces Proliferation and Migration in Breast Cancer Cells

**Fig. 3:**
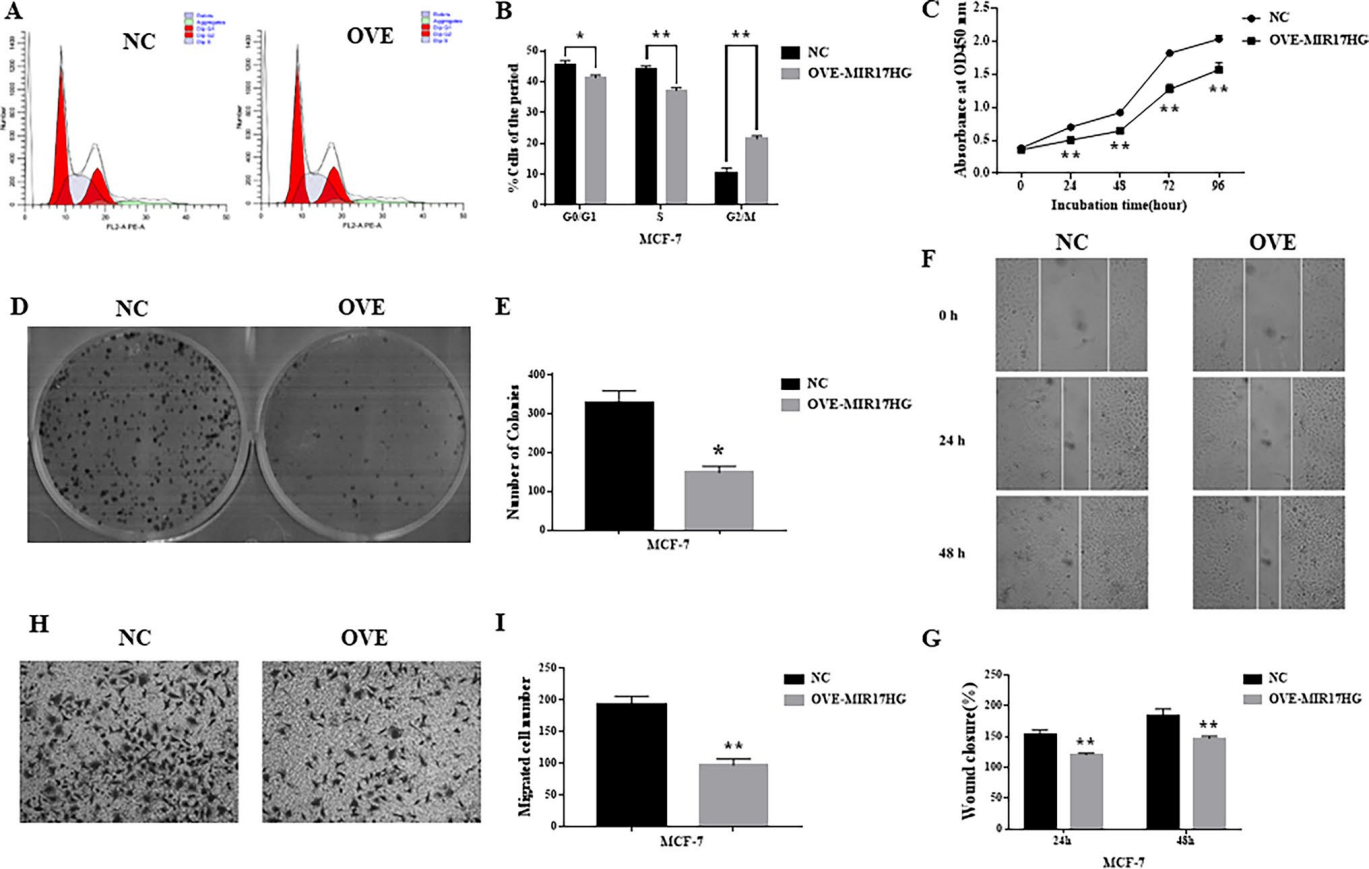
Overexpression of MIR17HG induces proliferation and migration in breast cancer cells. **A**, **B** Flow cytometry assay shows that Overexpression of MIR17HG results in G2/M arrest in breast cancer cells. The cell cycle distribution was exhibited. **C** Growth curves of MCF-7 cells after transfection with OVE-MIR17HG or NC is determined via CCK-8 assays. **D**, **E** Colony formation assays show that overexpression of MIR17HG inhibits breast cancer cell proliferation. **F**, **G** OVE-MIR17HG results in a slower closing of scratch wound in MCF-7 cells by wound healing assay. **H**, **I** Transwell migration assay is measured and the results are expressed as the number of invaded cells per field

To learn more about how MIR17HG overexpression affects breast cancer cells, MIR17HG is overexpressed in MCF-7 breast cancer cells, and the efficiency of overexpression is evaluated by qPCR, suggesting that MIR17HG overexpression is effective (Fig. 2I). MIR17HG overexpression increases the fraction of MCF-7 cells in the G2/M phase while decreasing the amount of cells in the G1 phase (Fig. 3A, B). These data suggest that MIR17HG overexpression causes MCF-7 cells to enter G2/M arrest, resulting in decreased cell proliferation. Following that, CCK8 assays are carried out. MIR17HG overexpression, according to our findings, substantially inhibits MCF-7 cell proliferation (Fig. 3C). Experiments on colony formation reveal that overexpression of MIR17HG reduces the number of colonies substantially, which is consistent with earlier experimental results (Fig. 3D, E). Then, using wound healing assays, we show that MIR17HG overexpression reduces breast cancer cells’ ability to migrate, which is consistent with our prior experimental findings (Fig. 3F, G). The effect of MIR17HG on the ability of breast cancer cells to migrate is next assessed using transwell assays. The results show that MIR17HG overexpression, according to the data, significantly reduces MCF-7 migration (Fig. 3H, I).

### MIR17HG Modulates FAM135A Expression by Sponging miR-454-3p

**Fig. 4:**
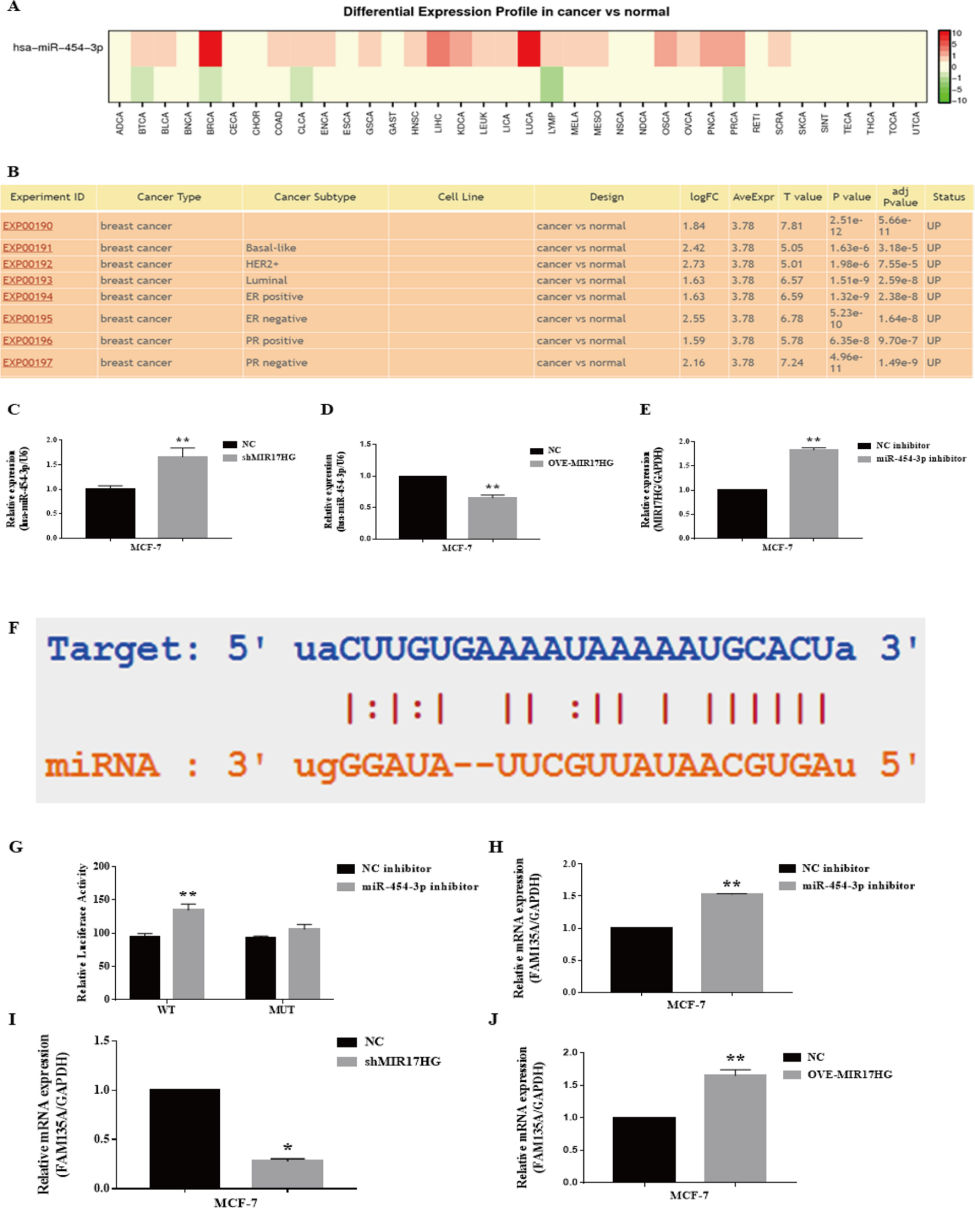
MIR17HG modulates FAM135A expression by sponging miR-454-3p. Search the expression of miR-454-3p in tumors (**A**), especially breast cancer (**B**) in dbDEMC 2.0. (**C**) The relative mRNA expression level of miR-454-3p is detected by qRT-PCR in MCF-7 cells transfected with shMIR17HG and negative control (NC). (**D**) The relative mRNA expression level of miR-454-3p is detected by qRT-PCR in MCF-7 cells transfected with OVE-MIR17HG and negative control (NC). (**E**) The relative mRNA expression level of MIR17HG is detected by qRT-PCR in MCF-7 cells transfected with miR-454-3p inhibitor and negative control inhibitor (NC inhibitor). (**F**) The predicted binding sites and mutated sites of miR-454-3p to the FAM135A sequence were shown. (**G**) Luciferase activity of HEK293 T cells cotransfected with miR-454-3p inhibitor, NC inhibitor and luciferase reporters containing FAM135A 3′UTR or FAM135A 3′UTR mut transcript were analyzed. (**H**) The relative mRNA expression level of FAM135A is detected by qRT-PCR in MCF-7 cells transfected with miR-454-3p inhibitor and negative control inhibitor (NC inhibitor). (**I**) The relative mRNA expression level of FAM135A is detected by qRT-PCR in MCF-7 cells transfected with shMIR17HG and negative control (NC). (**J**) The relative mRNA expression level of FAM135A is detected by qRT-PCR in MCF-7 cells transfected with OVE-MIR17HG and negative control (NC)

The ceRNA mechanism of lncRNAs has been widely explored. Thus, we performed bioinformatics analysis to predict probable MIR17HG targets using StarBase. miRNA miR-454-3p was later revealed to be related to MIR17HG. We have a database of miRNAs differentially expressed in human malignancies (version 2.0) dbDEMC 2.0 (https://www.picb.ac.cn/dbDEMC/index.html) to check up the expression of miR-454-3p in breast cancer, and identify miR-454-3p in biliary tract cancer, colon cancer, colorectal cancer, endometrial cancer, gastric cancer, head and neck squamous cell carcinoma, hepatocellular carcinoma, kidney cancer, leukemia, liver cancer, lung cancer, lymphoma, melanoma, mesothelioma, osteosarcoma, ovarian cancer, pancreatic cancer, prostate cancer, sarcoma and all subtypes of breast cancer are highly expressed (Fig. 4A, B). In MCF-7 cells, we observed that knockdown of MIR17HG increased miR-454-3p expression while suppressing FAM135A expression (Fig. 4C), and overexpression of MIR17HG suppressed miR-454-3p expression. (Fig. 4D) MIR17HG and FAM135A expression have also been shown to rise following transfection of MCF-7 cells with a miR-454-3p inhibitor (Fig. 4E, J). We postulated that MIR17HG and miR-454-3p regulate FAM135A expression via the ceRNA mechanism based on our findings and bioinformatics research. According to bioinformatics studies, miR-454-3p and FAM135A may have binding sites (Fig. 4F). To test their interaction, we created FAM135A reporter gene plasmids as well as mutant plasmids with altered binding sites. The miR-454-3p inhibitor increased the activity of FAM135A reporter gene plasmids while having no effect on mutant plasmids, according to reporter gene assays (Fig. 4I). Thus, our results imply that MIR17HG may operate as a ceRNA to upregulate FAM135A expression by sponging miR-454-3p.

### MIR17HG Induces Breast Cancer Growth and Migration Through Sponging miR‐454‐3p from FAM135A mRNA

**Fig. 5:**
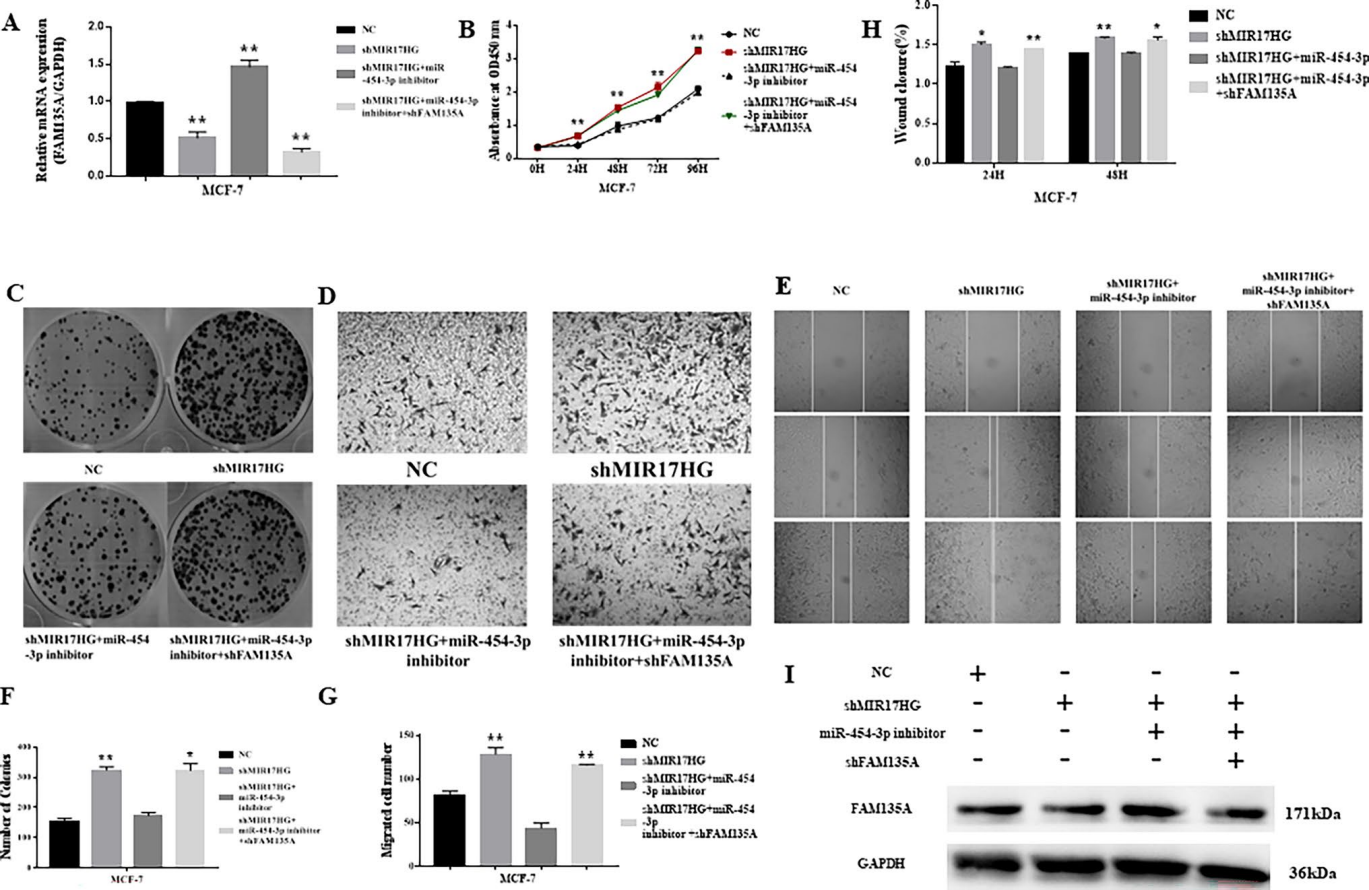
MIR17HG induces breast cancer growth and migration through sponging miR‐454‐3p from FAM135A mRNA. Rescue experiment is conducted. The expression level of FAM135A is detected by qRT-PCR (**A**) and western blotting (**I**) in MCF-7 cells transfected respectively with NC, shMIR17HG, shMIR17HG + miR-454-3p inhibitor and shMIR17HG + miR-454-3p inhibitor + shFAM135A. MIR17HG inhibits the colony (**C**, **F**) and proliferation (**B**, **D**, **G**) of breast cancer cells through the effect of miR-454-3p on FAM135A, MIR17HG inhibits the two-dimensional migration ability of breast cancer cells through the effect of miR-454-3p on FAM135A (**E**, **H**) MIR17HG inhibits the three-dimensional migration ability of breast cancer cells through the effect of miR-454-3p on FAM135A

The rescue tests are carried out to see if MIR17HG has a biological role in breast cancer cells via miR-454-3p. We utilize NC, shMIR17HG, shMIR17HG + miR-454-3p inhibitor, and shMIR17HG + miR-454-3p inhibitor + shFAM135A in that order for transient transfection. First, the effectiveness of transfection is confirmed by qRT-PCR and western blotting, suggesting that miR-454-3p mediates MIR17HG’s regulatory effect on FAM135A (See Fig. 5A and I). According to the results of the CCK8 assays, colony formation assay, Transwell assays, and wound healing assays, MIR17HG knockdown can significantly promotes MCF-7 breast cancer cell proliferation and migration, whereas miR-454-3p inhibitor can significantly inhibit MCF-7 cells proliferation and migration. Finally, when coupled with MIR17HG knockdown and a miR-454-3p inhibitor, shFAM135A substantially reduced MCF-7 cell growth and migration (Fig. 5B–H). These results suggest that MIR17HG, miR-454-3p, and FAM135A are part of an interacting regulatory network that influences breast cancer cell proliferation and migration.

### FAM135A is Linked to Breast Cancer Clinical Features as Well as a Number of Tumor-Related Signaling Pathways

**Fig. 6:**
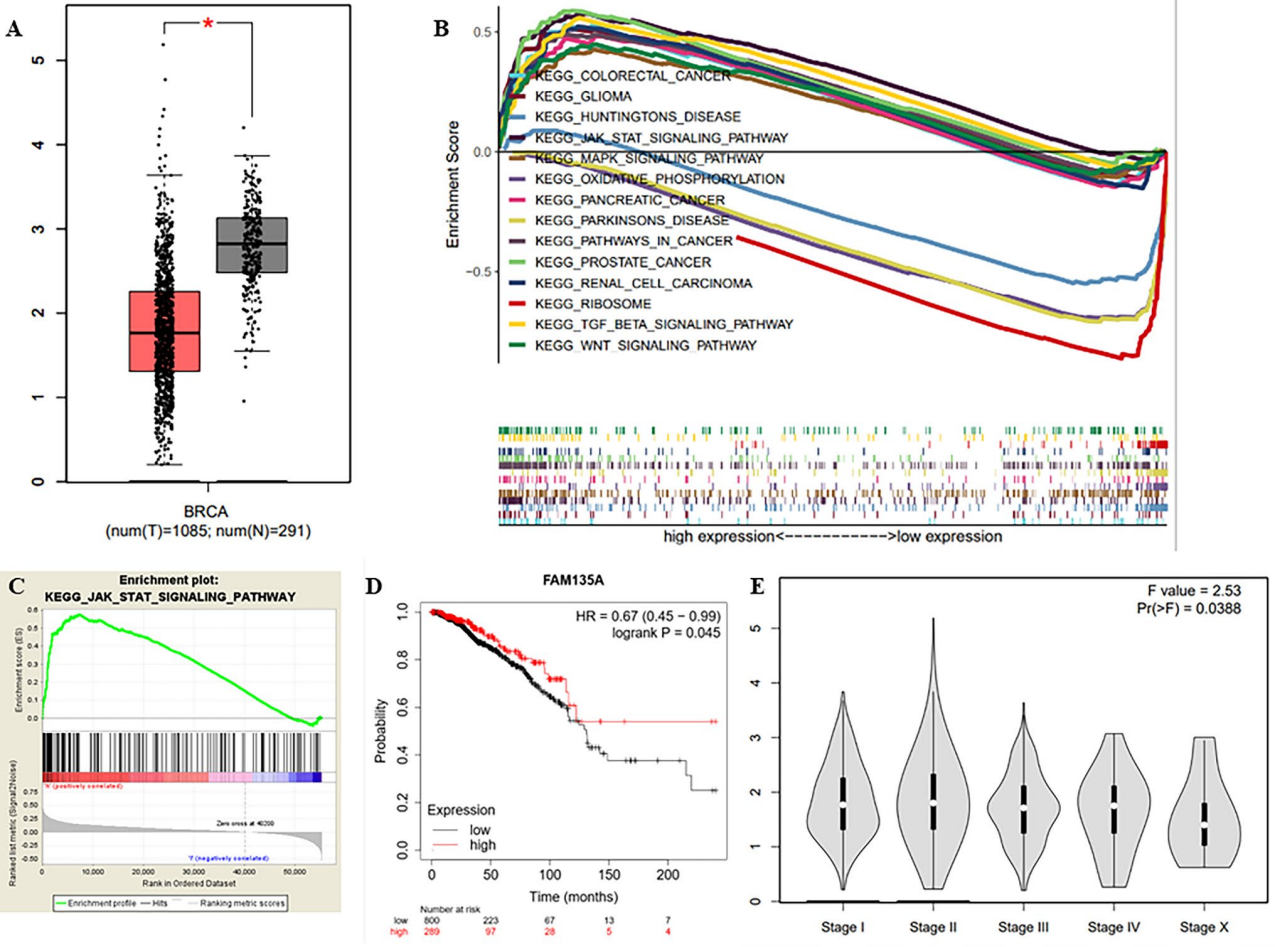
FAM135A is closely related to the clinical characteristics of breast cancer and a variety of tumor-related signaling pathways. **A** The differential expression of FAM135A in breast cancer samples and adjacent normal breast tissues is shown, according to TCGA database. **B** Pathways in cancer and their related KEGG pathways. **C** GSEA analysis revealed that the genes associated with FAM135A were mainly enriched in JAK-STAT signal related pathways. **D** FAM135A with survival percents of breast cancer patients is analyzed by Kaplan–Meier survival analysis. **E** The differential expression of FAM135A in different stages of breast cancer samples in TCGA database

FAM135A expression was lower in breast cancer tissues than in normal tissues adjacent (Fig. 6A). Furthermore, Kaplan–Meier survival analysis indicates that FAM135A expression is favorably associated with prognosis and negatively associated with stage in patients with breast cancer (Fig. 6D, E). According to GSEA, the expression of FAM135A is positively correlated to the pathways of colorectal cancer, glioma, JAK-STAT signal, MAPK signal, pancreatic cancer, cancer, prostate cancer, renal cell carcinoma, TGF-BETA signal pathway, WNT signal, and negatively correlated to the pathways of Huntington’s disease, Parkinson’s disease, oxidative phosphorylation, and ribosome, as shown in Fig. 6B. GSEA analysis revealed that the genes associated with FAM135A were mainly enriched in JAK-STAT signal related pathways (Fig. 6C). These findings indicate that FAM135A is linked to breast cancer clinical features as well as a number of tumor-related signal pathways, especially JAK-STAT signal pathways.

### Knockdown of FAM135A can Promote Proliferation and Metastasis in Breast Cancer Cells

**Fig. 7:**
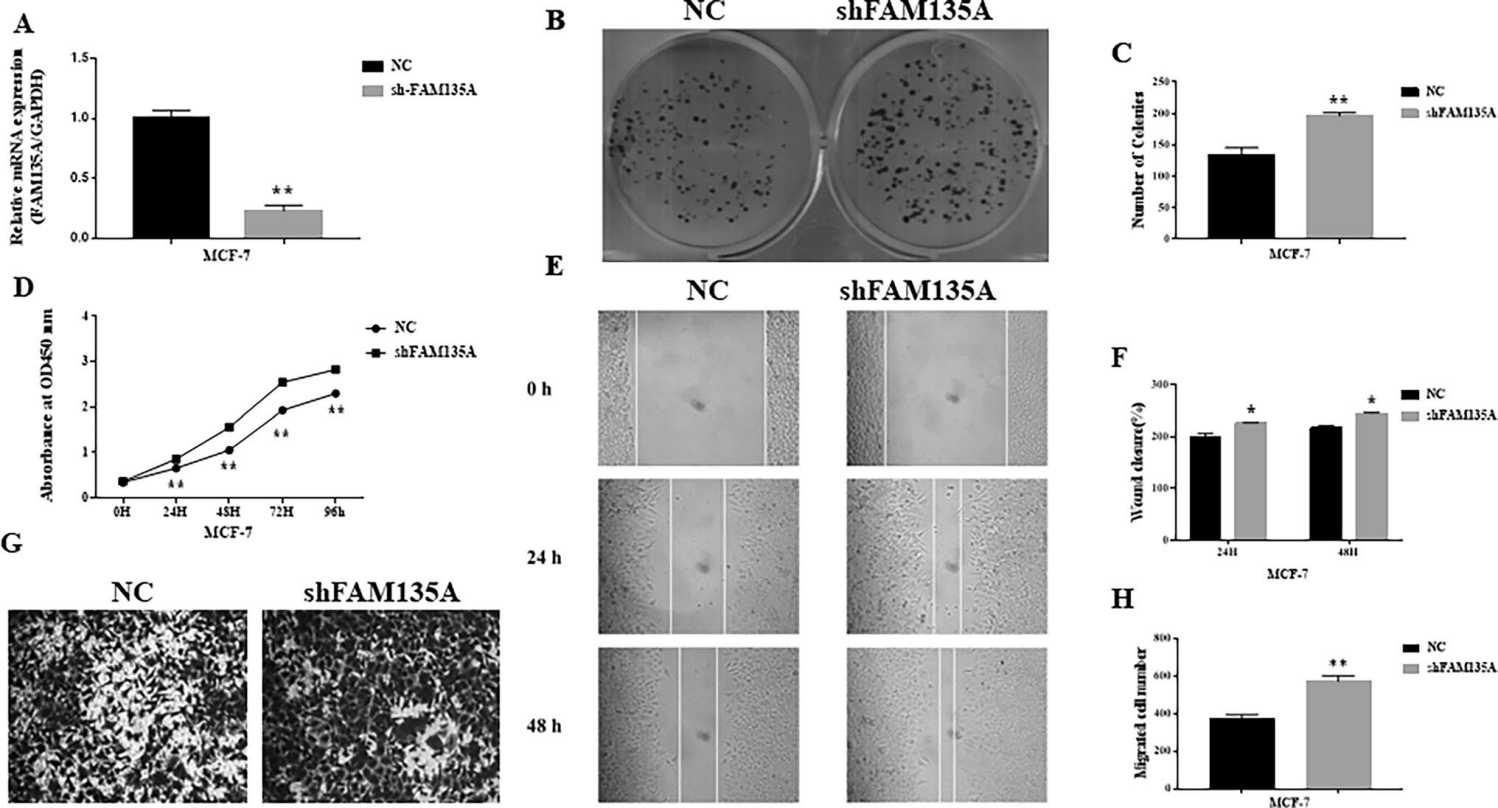
Knockdown of FAM135A can promote proliferation and metastasis in breast cancer cells. **A** The expression of FAM135A is significantly decreased by transfection of FAM135A shRNA in MCF-7 cells, which is detected by qRT-PCR. **B**, **C** Colony formation assays show that knockdown of FAM135A inhibits breast cancer cell proliferation. **D** Growth curves of MCF-7 cells after transfection with sh-FAM135A or sh-NC is determined via CCK-8 assays. **E**, **F** shFAM135A results in a quicker closing of scratch wound in MCF-7 cells by wound healing assay. **G**, **H** Transwell migration assay is measured and the results are expressed as the number of invaded cells per field

The knockdown effectiveness of shRNA designed to knock down FAM135A in breast cancer cells is evaluated using qPCR, which reveals that FAM135A shRNA is successful (Fig. 7A). CCK8 assays are performed when FAM135A is knocked down in MCF-7 cells. We find FAM135A knockdown significantly improves MCF-7 cell growth (Fig. 7D). Colony formation studies show that knocking down FAM135A increases the number of colonies, which is consistent with CCK8 assay results (Fig. 7B, C). Wound healing assays are performed and showed that wound closure occurred in MCF-7 transfected with shFAM135A as well as in the NC group, but wound closure is more pronounced in the former group (Fig. 7E, F). Using Transwell assays, we then look at how FAM135A affects breast cancer cell migration. FAM135A downregulation, according to the data, significantly aided MCF-7 migration (Fig. 7G, H).

## Discussion

Breast cancer is the most prevalent cancer in women worldwide, and it is one of the leading causes of cancer deaths in women [20]. Long non-coding RNA (lncRNA) is a kind of non-coding RNA that plays an important role in carcinogenesis and is engaged in normal human body development [21].

More and more evidence suggests that lncRNA functions in a variety of biological processes in the body and is a crucial regulator in human illnesses [22]. Through a multitude of regulatory mechanisms, it governs the body’s proliferation, apoptosis, and even cancer. Lnc015192 regulates miR-34a and subsequently targets Adam12 in breast cancer through a ceRNA mechanism [23], and studies have also shown that LncRNA BLACAT1 promotes glioma development by binding to miR-605-3p to regulate VASP expression and promote glioma development and progression [24]. MIR17HG, a breast cancer-related lncRNA, was shown to be considerably down-regulated in breast cancer tissues and cells in this study, and its low expression was linked to advanced TNM stage, tumor volume expansion, and survival rate. Colorectal cancer [13, 25], gastric cancer [15], glioma [26, 27], and other tumor types are currently the subject of MIR17HG reports. We find lower lncRNA MIR17HG expression is associated with a worse prognosis in breast cancer, according to our findings. To investigate MIR17HG’s role in breast cancer, we used shRNA technology to knock it down in MCF-7 cells. MIR17HG knockdown increases breast cancer cell proliferation in vitro, according to CCK8 assays and clone creation studies. In transwell experiments, MIR17HG knockdown was linked to enhanced breast cancer cell migration. Furthermore, we find that overexpression of MIR17HG has a substantial effect on the cell cycle of MCF-7 cells, which validates that our earlier findings. The percentage of MCF-7 in the G2/M phase increases when MIR17HG is overexpressed. MIR17HG is a tumor suppressor gene in breast cancer, according to our results.

Tumor emergence and progression is a complex process involving several variables, genes, and stages of change [22]. The biological implications and mechanism of non-coding RNA are revealed as more study is conducted. There are studies which have shown that lncRNA can directly regulate the expression of neighboring genes through the cis‐acting mechanism [4, 10, 28], which is a direct and one‐way regulation method. However, the regulation between lncRNA and target genes in this regulation mode is indirect and mutual. The “ceRNA theory” is one of them. The underlying idea is that the cell has a competing endogenous RNA (ceRNA). These ceRNA molecules, including mRNAs, lncRNAs and others, can bind the same miRNAs through miRNA response elements (MREs), thereby regulating their respective production levels [23]. According to a growing body of evidence, MIR17HG may interact with the corresponding mRNA via the ceRNA mechanism in many cancers such as colorectal cancer and gastric cancer [8]. As a result, we believe MIR17HG may have a similar effect in breast cancer. miRNA attaches directly to the target gene’s 3′untranslated region (3′-UTR region), perhaps causing further gene degradation or inhibiting protein translation. We previously used bioinformatics analysis on the StarBase to determine the likely target of MIR17HG. Finally, miR-454-3p was discovered to be linked to MIR17HG. The presence of miR-454-3p in tumors has been demonstrated, and it is assumed to play an important role in cancer formation. miR-454-3p levels have been found to be downregulated in gliomas [24], and miR-454-3p promotes the development of non-small cell carcinoma. Linc00887 inhibits the development of cervical cancer via activating the FRMD6-Hippo signal pathway and competitively binds miR-454-3p in cervical cancer [25]. Overexpression of miR-454-3p has been linked to a shorter recurrence-free survival in breast cancer patients and has been shown to cause in vivo breast cancer metastasis, suggesting that it might be a potential diagnostic and therapeutic target for breast cancer metastasis [26]. We look for the expression of miR-454-3p in breast cancer in the dbDEMC 2.0. We discover that miR-454-3p is significantly overexpressed in biliary tract cancer, colon cancer, colorectal cancer, endometrial cancer, gastric cancer, head and neck squamous cell carcinoma, hepatocellular carcinoma, kidney cancer, leukemia, liver cancer, lung cancer, lymphoma, melanoma, mesothelioma, osteosarcoma, ovarian cancer, pancreatic cancer, prostate cancer, sarcoma and all subtypes of breast cancer.

We also used the assay to demonstrate that MIR17HG targets miR-454-3p. The miR-454-3p expression in breast cancer cells increases when MIR17HG is knocked down, and when MIR17HG is overexpressed, the amount of miR-454-3p expressed out to be decreasing in breast cancer cells. MIR17HG expression is enhanced in breast cancer cells when the miR-454-3p inhibitor is transfected into MCF-7 cells to reduce miR-454-3p expression. According to our findings, MIR17HG appears to be competitive for miR-454-3p in breast cancer.

Furthermore, we predict that FAM135A is the target gene of miR-454-3p using StarBase. According to our findings, MIR17HG may increase FAM135A expression by acting as a competitive binding sponge for miR-454-3p. MIR17HG knockdown reduces FAM135A mRNA expression in breast cancer cells, but MIR17HG overexpression enhances FAM135A mRNA expression. Little research on the FAM135A gene has been done. FAM135A is found to increase the proliferation and migration of breast cancer cells when it is down-regulated. Following transfection with the miR-454-3p inhibitor to reduce miR-454-3p expression, the mRNA expression level of FAM135A in breast cancer cells is enhanced. To examine their interaction, luciferase assays and the generation of wild-type (WT) and mutant (MUT) luciferase reporter genes are utilized. When compared to 293 T cells transfected with pMIR-report-FAM135A 3′UTR MUT, the fluorescein enzyme activity of 293 T cells transfected with pMIR-report-FAM135A 3′UTR WT increased substantially after transfection with miR-454-3p inhibitor. miR-454-3p inhibition promotes the activity of the FAM135A WT reporter gene in MCF-7 cells, indicating that miR-454-3p regulates the FAM135A 3′UTR region. Rescue experiments were also conducted to show that MIR17HG regulates FAM135A through miR-454-3p. These findings support the hypothesis that miR-454-3p acts as a mediator in the interaction between MIR17HG and FAM135A. The excessive proliferation of tumors is often accompanied by immune regulatory and cell cycle disorders. We used GSEA analysis to explore the function of real hub genes, and we found that FAM135A was significantly enriched in functions and pathways related to “JAK-STAT Signaling”. This gene was also enriched in the pathway related to “Cancer-Related”, and we speculate that FAM135A may play a key role in the pathogenesis of breast cancer through JAK-STAT signaling pathway. Janus kinase-signal transducer and activator of transcription (JAK-STAT) signaling mediates almost all immune regulatory processes, including those involved in tumor cell recognition and tumor-driven immune escape [29]. Notably, the JAK/STAT signaling cascade is an important regulatory pathway mediating breast tumor growth and survival [30]. There is evidence supporting a direct correlation between STAT3 activation and increased Cyclin D1 expression in primary breast tumors and breast cancer-derived cell lines 6, 56. Consistently, loss or depletion of STAT3 in breast carcinoma cells has been shown to result in tumor inhibition and induction of apoptosis [31]. Our findings imply that this axis might be a new treatment target for breast cancer. Given that the understanding of the mechanisms underlying cell proliferation and migration can provide crucial insights for the development of anticancer therapeutic agents, the identification of molecules that play an important role in cell motility is therefore imperative in the fight against cancer metastasis. Due to the novelty of MIR17HG, more studies need to be carried out to further understand the mechanisms by which it regulates breast tumorigenesis through modulating both miR-454-3p/FAM135A axis and JAK/STAT signaling. Nonetheless, our study suggests that MIR17HG is a key signaling molecule that functions to inhibit cell migration in breast cancer, providing the foundation for its development as a novel biomarker in breast tumors. In this study, we discovered that MIR17HG/miR-454-3p/FAM135A axis functions in preventing development and metastasis in breast cancer. These novel findings indicate that MIR17HG/miR-454-3p/FAM135A axis may be a potential target for breast cancer therapy to improve the survival time and quality of life of patients. Our findings reveal the function of MIR17HG/miR-454-3p/FAM135A axis in breast cancer and provide potential novel therapeutic targets for metastatic breast cancer intervention.

## Conclusion

Our findings demonstrated that MIR17HG might suppress breast cancer cell proliferation and migration by sponge miR-454-3p through ceRNA(competing endogenous RNAs) mechanism, indicating that targeting MIR17HG may be a feasible therapeutic candidate for breast cancer. Meaningfully, new gene therapy is an effective assistant method in reducing the harm of surgery on patients. More studies need to be carried out to further understand the mechanisms by which it regulates breast tumorigenesis through modulating both miR-454-3p/FAM135A axis and JAK/STAT signaling. We will explore whether MIR17HG should be considered a new candidate drug molecule for preclinical testing in our further study.

## Data Availability

The original contributions presented in the study are included in the article. Further inquiries can be directed to the corresponding author.
